# Formulation and Pharmacokinetic Evaluation of Microcapsules Containing Pravastatin Sodium Using Rats

**DOI:** 10.1155/2016/7623193

**Published:** 2016-08-09

**Authors:** Venkatesh Dinnekere Puttegowda, Roopa Karki, Divakar Goli, Sajal kumar Jha, Manjunatha Panduranga Mudagal

**Affiliations:** ^1^Department of Pharmaceutics & Pharmacology, Acharya and B. M. Reddy College of Pharmacy, Soldevanahalli, Bengaluru 560107, India; ^2^Department of Pharmaceutics, School of Pharmacy, Guru Nanak Institutions Technical Campus, Ibrahimpatnam, Telangana, India

## Abstract

Pravastatin Sodium has a cholesterol lowering agent. It has shorter half-life and undergoes first-pass metabolism. Frequent dose is required in case of conventional dosage form. The purpose of the study is to formulate and evaluate microcapsules containing Pravastatin Sodium by complex with cholestyramine resins coated with Eudragit RLPO and Eudragit RSPO polymers for achieving control release. Complexation of drug on resin was carried out by batch method. Microencapsulation was carried out by nonaqueous solvent evaporation method. Pharmacokinetic studies were done by using rats. The intermediate stability studies were carried out on the most satisfactory formulations. FTIR, X-ray diffraction, and DSC spectra of drug, drug-resinates, and polymers revealed no chemical interaction. The % DEE and % yield were observed for formulations of f1 to f7 that were varied from 97.1 ± 0.8 to 98.9 ± 0.5% and 95.0 ± 3.25 to 98.8 ± 7.1%, respectively. Most satisfactory formulation, f6, showed drug release up to 72.6%. No changes in % DEE and % CDR were observed after stability studies. Microcapsules of f6 formulation achieved best performance regarding* in vitro* drug release and from pharmacokinetic evaluation mean residence time was found to be 6.3 h, thus indicated, Pravastatin Sodium microcapsules were released and absorbed slowly over a prolonged period of time.

## 1. Introduction

Pravastatin Sodium is a class of lipid regulating drug, the statins which reduce cholesterol biosynthesis. This agent is competitive inhibitor of HMG CoA reductase. It is an odour-free, white to faded white, fine or crystalline powder. It is a moderately polar, hydrophilic compound. It is, to some extent, rapidly absorbed from the gastrointestinal tract and undertakes extensive first pass metabolism in the liver. The absolute bioavailability of Pravastatin Sodium is 17%. About 50% of the circulate drug is bound to plasma proteins. Plasma elimination half-life is 1.5 to 2 h. About 70% of oral dose is excreted in the feces and about 20% is excreted in the urine [1].

The more established bile acid sequestrants such as cholestyramine and colestipol are among the old hypolipidemic agents, and they are most likely the safest, as they are not absorbed from the intestine. Statins are not as useful as monotherapy; the resins are more frequently used as second agents, if the statin therapy does not lower LDL-C levels sufficiently. When used with cholestyramine and colestipol it is generally prescribed with submaximal doses [2]. The cholestyramine resins are highly positive charged and bind negatively charged bile acids; because of their bulky size, the resins were not absorbed and the bound bile acids are excreted in the feces. In the synthesis caused by upregulation of HMG CoA reductase enzyme activity by a statin considerably increases the effectiveness of the resins [3]. Controlled or sustained release property can also be imparted to oral dosage formulation through the development of resin drug complexes (drug-resinates). The drug is released from resin* in vivo* as the drug-resinates reach equilibrium with the more electrolyte concentration found in the gastrointestinal tract [4]. Resins bind bile acids in the intestine, in this manner interrupting the enterohepatic circulation of the bile acids, and increase the conversion of cholesterol into bile acid in the liver [5].

Ion exchange resins have been used widely in the pharmaceutical industry as drug delivery system. The drug release rate of ion exchange resins can be modified depending on functional group, ion exchange capacity, degree of cross-link, and particle size. Ion exchange resins have fixed ionic functional groups which can provide binding of ionic drugs. Release of the bound drug requires exchange with counter ions such as hydrogen or sodium, which are available in the gastrointestinal tract. The overall drug release kinetics of polymer coated ion exchange resins is mainly dependent upon the drug and counter ion, diffusion confrontation in the coating film and boundary layer (film) surrounding the particles, surrounding medium, liquid diffusion, and drug dissolution [6]. Controlled or sustained release properties can also be impaired to oral dosage formulations through the formation of drug-resinate complex. The drug is released from the resin* in vivo* as the drug-resinates reach equilibrium with the high electrolyte concentrations typically found in the gastrointestinal tract [5].

When used as an active ingredient, cholestyramine resins are a strong pharmacological bile salt-binding agent that binds bile acid; this leads to replenishment of the bile acids through increasing the catabolism of serum cholesterol resulting in lowered serum cholesterol levels. Bile acids are the end products of cholesterol metabolism. They are synthesized in liver and secrete bile into the intestine, where they aid in the absorption of fat soluble vitamins and dietary fat. Subsequently bile acids come again to the liver to complete their enterohepatic circulation. Resins bind bile acids in the intestine, thereby interrupting the enterohepatic circulation of the bile acids and increasing the renovation of cholesterol into bile acids in the liver [7]. Positively charged anion resins bind negatively charged bile acids, about 95% of which are normally reabsorbed. Drug release from the resins depends on two factors, the ionic environment, that is, pH and electrolyte concentration within the gastrointestinal tract, and the properties of the resin [7].

The cholestyramine resin is an insoluble, strongly basic anion exchange resin in the chloride form supplied as dry fine powder. Cholestyramine resins Tulsion 412 was used as a drug carrier for anionic drug Pravastatin Sodium. Ion exchange resins alone without any barrier cannot achieve satisfactory controlled release. Thus resin complexes were coated with polymers for achieving controlled release in the small intestine. Microencapsulation is an effective method to wrap liquid or solid materials which are surrounded by coating with polymeric membrane. The purpose of the study is to formulate microcapsules by complexing Pravastatin Sodium drug with anionic exchange resins and further coat with Eudragit RLPO and Eudragit RSPO polymers for achieving controlled release in the small intestine.

## 2. Materials and Methods

### 2.1. Materials

Pravastatin Sodium gift sample was collected from Biocon India Ltd, Bengaluru. Tulsion 412 resins collected were gift from Thermax ion exchange Mumbai, India. Eudragit RSPO and Eudragit RLPO polymers gift samples were collected from Evonik's laboratory Mumbai; Span 80 was collected from Karnataka fine chemicals Bengaluru and all other chemicals and solvents used were of analytical grade.

### 2.2. Drug Polymer Compatibility Studies

Drug polymer interaction studies were carried out by using FTIR, DSC, and X-ray diffraction studies. FTIR spectroscopy is a powerful tool for identifying types of chemical bonds and functional groups and checks the integrity of drug in the formulation. In the present study, a pinch of pure drug was placed in the spectrophotometer and the spectrum was recorded. The characteristic peaks of the pure drug and drug peaks from the formulations were identified. It was performed by FTIR over frequency range 4000–400 cm^−1^ using Bruker Tensor 27 Germany.

XRD is required in drug development to assess the state, that is, amorphous or crystalline nature, of the drug and degree of crystallinity of particular substances. Pravastatin Sodium, resins, and drug resin complex were analysed by X-ray powder diffraction. All the samples were run at (2*θ*) min^−1^ from 10° to 60° (2*θ*). The XRD patterns of drug, resins, and drug-resinates were recorded and compared.

Thermal analytical methods used in the characterization of polymorphs, purity and compatibility studies of the sample. Differential scanning calorimeter was used to study thermal transitions of a drug, resins, and drug resin complex. In DSC, thermogram was obtained at a heating rate 10°C/min, over a temperature range 35–500°C. The samples were hermetically sealed in an aluminium crucible. Nitrogen gases were purged at a rate of 10 mL/min, for maintaining inert atmosphere. The instrument was calibrated for temperature and heat flow using indium and zinc standards, respectively.

### 2.3. Preparation of Drug-Resinates

Preparation of drug-resinates was tried by batch method [5]. Accurately weighed drug and cholestyramine resins were in 1 : 4 ratios. Then slurry of resin was made in 100 mL distilled water and stirred half an hour at 500 rpm, to allow the polymer structure to swell uniformly. Drug solution in distilled water was slowly added to resin slurry under mixing condition, neutral pH 7 was maintained because at neutral pH maximum drug load was achieved. The mixture was stirred for 5 h continuously on magnetic stirrer. Drug loading of Pravastatin Sodium was determined spectrophotometrically at 238.5 nm.

### 2.4. Determination of Amount of Uncomplexed Drug by UV

The mixture was kept aside to allow the particles to sediment and then filtered. From the filtrate 1 mL was transferred into 10 mL volumetric flask; after suitable dilution, drug was determined spectrophotometrically at 238.5 nm. The amount of drug loading in percentage was calculated [6].

### 2.5. Preparation of Microcapsules

Preparations of microcapsules were done as per formulation chart given in Table 1. Microcapsules were prepared by using solvent evaporation technique [8]. A homogenous polymer solution was made in 20 mL acetone in a beaker. Drug-resinates were dispersed uniformly into 70 mL liquid paraffin containing one drop of Span 80 in another beaker. It was stirred by using mechanical stirrer at speed of 500 rpm. Polymer solution was injected slowly using 20-gauge syringe under stirred condition continuously for 3 to 4 h until acetone was evaporated. The microcapsules were collected by using vacuum filtration followed by washing 4-5 times with petroleum ether and dried at room temperature for 24 h.

### 2.6. Evaluation of Microcapsules

#### 2.6.1. Microscopic Observation

Particle size and shape of microcapsules were observed using compound microscope.

#### 2.6.2. Yield of Microcapsules

The prepared microcapsules were collected and weighed. Yield of microcapsules was calculated by actual weight of microcapsules divided by total weight of copolymers and drug:

Percentage yield = (Actual weight of microcapsules/Total weight of copolymers and drug) × 100.

#### 2.6.3. Scanning Electron Microscopy

Microcapsules were pragmatic under scanning electron microscope (JEOL 5400, Tokyo, Japan) for surface characteristics and examine the morphology of fracture surface. The sample was dried and coated with a gold ion for 5 to 6 min to obtain 15 KV using SE^2^ detector at various magnifications [9].

#### 2.6.4. Micromeritic Properties

Flow properties of the microcapsules were evaluated by determining the angle of repose and the compressibility index. Angle of repose was calculated by fixed funnel method. Bulk density was measured by using bulk density apparatus and compressibility index was calculated.

#### 2.6.5. Particle Size Analysis

Particle Size analysis was determined using compound microscope with the help of stage micrometer and eye piece micrometer, counting at least 100 microcapsules per batch. Microcapsules were counted at 10x magnification.

#### 2.6.6. Percentage Drug Entrapment Efficiency

Accurately weighed microcapsules equivalent to 10 mg of drug were stirred with 100 mL of pH 6.8 buffer for 2 hours. This solution was filtered and after suitable dilution drug content was estimated spectrophotometrically at 238.5 nm. One has the following:

Percentage drug efficiency = (Amount of drug actually present/theoretical drug load expected) × 100.

#### 2.6.7. Determination of Coating Polymer on Microcapsules

About 1 g of the microcapsules was accurately weighed and washed 3 times with 10 mL of acetone in order to remove polymer coating. The remaining drug-resinate core was dried at 50°C for 12 h and weighed. The percentage of coating polymer was calculated by the following equation:

Percentage coating polymer = (Microcapsules weight − Dried complex weight/Microcapsules weight) × 100.

#### 2.6.8. *In Vitro* Drug Release Study

Microcapsules equivalent to 40 mg of the drug were used for dissolution study. USP type I dissolution test apparatus was used. The dissolution tests were carried out using 870 mL of pH 1.2 phosphate buffer for first 2 h and made up to 900 mL by adjusting the pH 6.8 phosphate buffer for remaining 6 h, a speed of 50 rpm, and temperature 37 ± 0.5°C. The amount of dissolved drug was determined using UV spectrophotometric method at 238.5 nm.

#### 2.6.9. Pharmacokinetic Study Design and Protocol

*In vivo* study design and protocol were approved by institutional ethical committee. Twenty healthy rats of either sex, weighing 200–220 g, were used for the study; test, standard, and control, in each group (*n* = 6) rats, were used. A crossover experimental design with a washout period of 1 month was followed for testing the formulation. Rats were kept for overnight fasting. Only water was permitted until 24 h following oral administration of formulation. Microcapsules were suspended in 0.1% Carboxy methyl cellulose solution and sonicated for 5 min to get uniform dispersion. Rat dose was calculated based on the weight of the rats. Blood samples were collected from retroorbital plexus vein from rat eye. After collecting the “zero” hour blood sample (blank), 1 mL sample was administered orally using rat feeding tube. About two mL of blood samples was collected 0.5, 1, 2, 4, 6, 8, 12, and 24 h at each time interval after administration. The blood samples were centrifuged at 6000 rpm and serum separated was collected into dry test tubes and all the samples were stored under defreeze maintained temperature −40°C. Pravastatin Sodium drug from serum concentrations was determined by a HPLC method as follows: methanol 1 mL was added to 0.5 mL of serum and agitated for cyclomix for 2 to 3 min followed by using cooling centrifuge at 4°C maintained speed 5000 rpm for 10 minutes. The clear liquid was removed and placed in Eppendorf tube. Then the supernatant was diluted with mobile phase Acetonitrile: 1 octane sulphonic acid at pH 2.5: 60 : 40, flow rate 1 mL/min, injection volume 20 *μ*L of solution injected in to HPLC column (column) dimension ID: 250 × 4.6 mm, particle size: 5 *μ*m, and high pressure gradient, detector: UV wavelength: 230 nm.

From the time v/s serum drug concentration data various pharmacokinetic parameters such as peak plasma concentration (*C*_max_), time at which peak occurred (*t*_max_), area under the curve, elimination rate constant (*K*_el_), biological half-life (*t*_1/2_), absorption rate constant, and *V*_*d*_ were calculated as per known calculation methods. Highest concentration of drug in plasma attained by the administrated dose is *C*_max_. Time taken to reach maximum concentration of drug in plasma is *t*_max_. Area under the curve was calculated by using trapezoidal rule. *V*_*d*_ was calculated by using formula: Administrated dose/Initial plasma drug concentration. Biological half-life was calculated by using formula 0.693/ke [10].

Absorption rate constant was calculated by using method of residuals. Clearance (Cl) was calculated by using formula: Administered dose/AUC. Mean residence time was calculated based on 63.2% of drug eliminated from the body [7].

#### 2.6.10. Statistical Analysis

Student's paired *t*-test was used for statistical evaluation of the results of two groups. *P* value of <0.05 was considered to represent a statistical significant difference.

#### 2.6.11. Stability Studies

The intermediate stability studies were carried out on the most satisfactory formulations according to ICH guidelines. The formulations were sealed in aluminium packaging and kept in stability chamber maintained at 30 ± 2°C/65% RH for six months.

## 3. Results and Discussion

**Figure 1:**
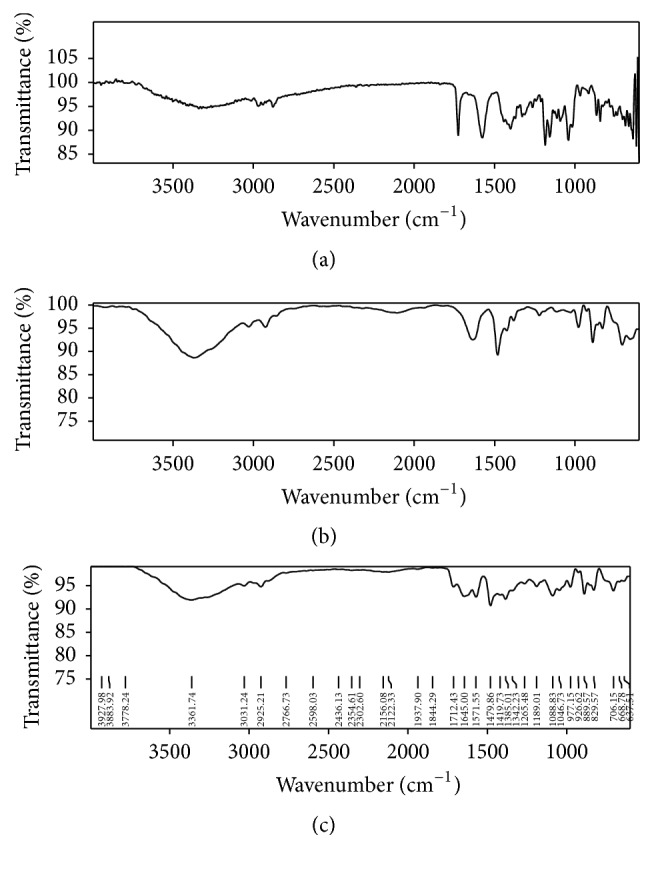
FTIR Spectra of (a) Pravastatin Sodium, (b) Tulsion 412, and (c) drug-resinates.

**Figure 2:**
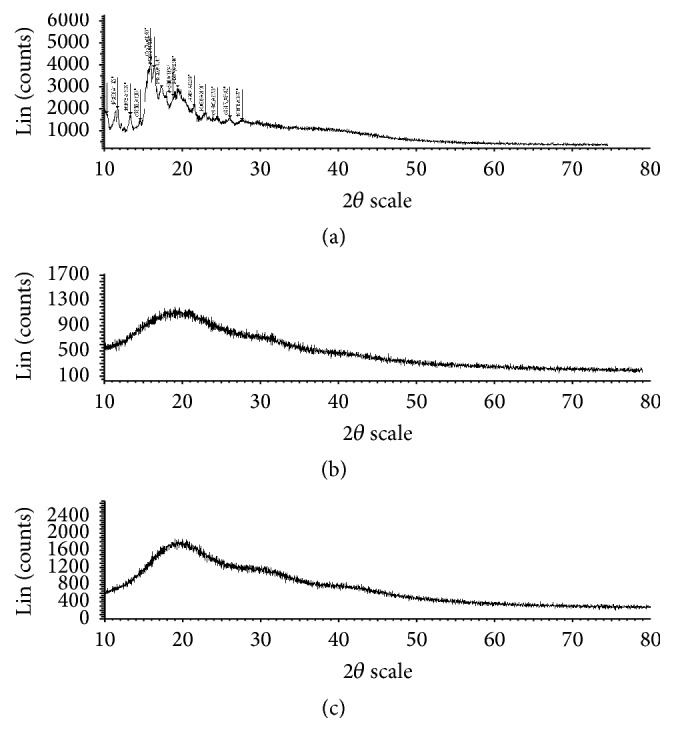
X-ray diffraction spectra of (a) Pravastatin Sodium, (b) Tulsion 412, and (c) drug-resinates.

**Figure 3:**
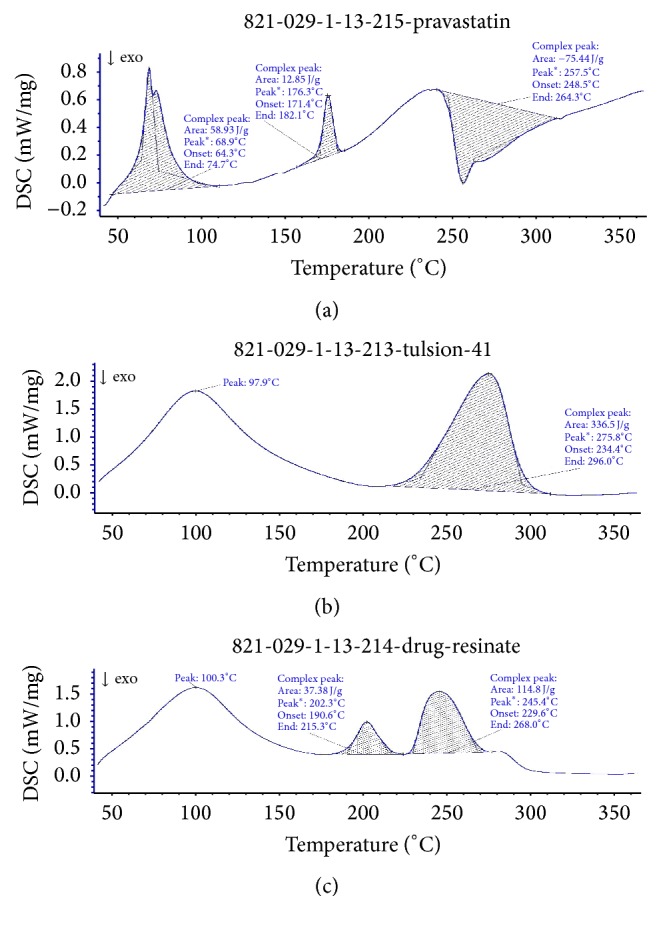
DSC spectra of (a) Pravastatin Sodium, (b) Tulsion 412, and (c) drug-resinates.

**Figure 4:**
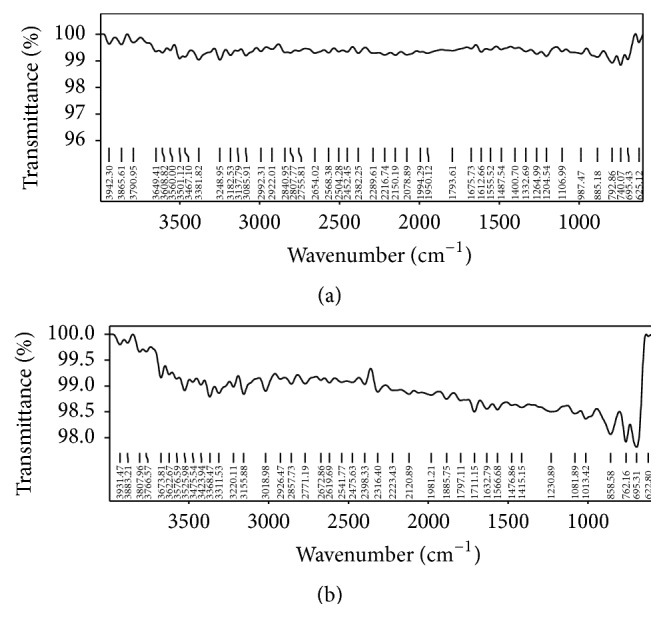
FTIR spectra of Eudragit RLPO coated microcapsules and Eudragit RSPO coated microcapsules.

**Figure 5:**
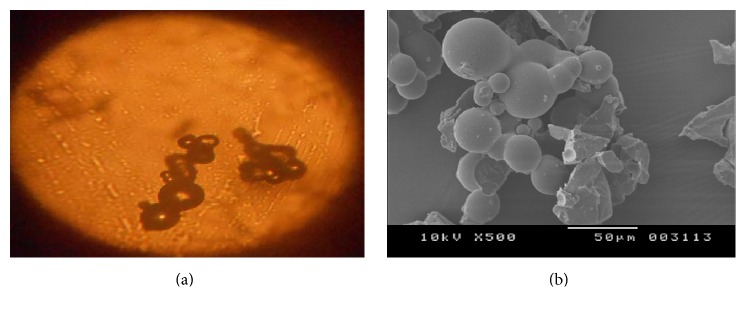
(a) Microscopic images at 10x magnification and (b) SEM images of Eudragit RSPO microcapsules.

Based on the solubility 0.1 N HCl and pH 6.8 phosphate buffer was used for constructing the standard graph of Pravastatin Sodium and same medium was used for dissolution studies. Batch method was used for complexation of drug with resins. Maximum drug load was observed in 1 : 4 ratios. FTIR spectrum of Pravastatin Sodium peaks has C=O at 1712 cm^−1^, OH at 3361.74 cm^−1^, 3031 cm^−1^ due to SP3 CH stretching, 1479 cm^−1^ due to CH bending alkanes, and SP2-CH stretching, which are characteristic peaks of pure drug Pravastatin Sodium; after complexation with resins same considerable peak was observed as shown in the Figure 1. The X-ray diffraction of drug alone shows sharp crystalline peaks after complex with resins most diffused peaks were observed as showed in Figure 2. DSC spectra of pure drug exhibited sharp exothermic peak at 68.9°C and 176°C and endothermic peak at 257.5°C. Drug-resinates peaks are deviated due to complexation as shown in Figure 3. The preparation of drug-resinate was optimized with respect to drug resin ratio and time of sorption. Drug loading was done by batch method with cholestyramine anion exchange resin Tulsion 412. It was stirred at 4 h continuously in which optimum drug load 80.34% was experimented in 1 : 4 ratio; even if we increase the drug resin ratios, there was no changes in drug loading. Further batches were prepared by using same 1 : 4 ratios. Microcapsules were prepared by nonaqueous solvent evaporation technique. Drug characteristic peaks were observed for prepared microcapsules identified from FTIR spectra of Eudragit RLPO coated microcapsules and RSPO coated microcapsules as shown in Figure 4. Morphology of microcapsules was observed from compound microscope and SEM. Scanning electron micrographs of single and group of Eudragit RLPO and Eudragit RSPO microcapsules were observed. The micro particles were irregular shaped, free flowing powders in case of RLPO microcapsules. Spherical, discrete, and number of microporous surface structure was observed in case of Eudragit RSPO microcapsules as shown in Figure 5. Physicochemical parameters of all f1 to f7 formulations were showed in Table 2. Microcapsules were free flowing powders. Angle of repose was measured by using fixed funnel method. The angle of repose was observed in the range of 22.0° to 23.5°; it fulfils the IP requirements. Bulk density was found to be 0.48 to 0.68 g/cc. The lower values of bulk density were favourable for obtaining higher porosity. Compressibility index was found to be 5.00 to 7.50%. Values below 15% usually give rise to good flow and compressibility characteristics. Percentage yield for the batches f1 to f7 varied from 98.0 ± 2.2 to 98.9 ± 7.5%. Percentage drug entrapment efficiency for the formulations f1 to f7 varied from 97.0 ± 0.5% to 98.7 ± 0.3%. When the polymer coating increases, microcapsules particle size also increases as shown in Table 2.

**Figure 6:**
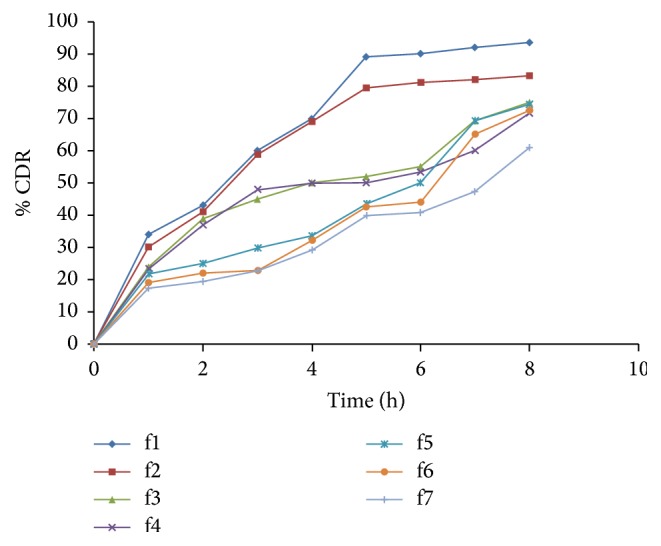
*In vitro* drug dissolution profiles of f1–f7.

The drug release in the acidic media (pH 1.2) is low because drug is weak acid and its pKa value is 4.2, but, in presence of alkali media (pH 6.8), drug discharge rate was faster due to phosphate ions. The drug release from the Eudragit RLPO and Eudragit RSPO coated microcapsules shows slower drug release than uncoated resinates. Most satisfactory formulation f6 showed drug release up to 72.6% as shown in Figure 6. The drug release data were fitted into drug release kinetic models. It follows mixed order kinetics. Formulations f2 to f4 follow first-order kinetics and f5 to f7 follow zero-order kinetics observed based on the regression coefficient values as shown in Table 3. The uncoated resinates followed particle diffusion process. However coated resinate deviated from particle diffusion. Microcapsules follow membrane diffusion control mechanism in the drug release system. The data was fitted into Korsmeyer-Peppas equation. Based on the *n* values from 0.56 to 0.76, this indicates non-Fickian diffusion type of mechanism. The drug release data from Higuchi equation showed comparable linearity. Microcapsules obeyed diffusion controlled process. Based on release kinetics data microcapsules of f5 and f6 formulations endowed with a convenient dosage form for achieving controlled release. The f5 and f6 drug release data was expressed ± SD. Paired *t*-test was used for statistical evaluation of results. There were 8 samples for both the groups. *P* value < 0.05 was considered to represent a statistical significant difference. Results from dissolution data f5 and f6 were statistically significant for 5% level.

**Figure 7:**
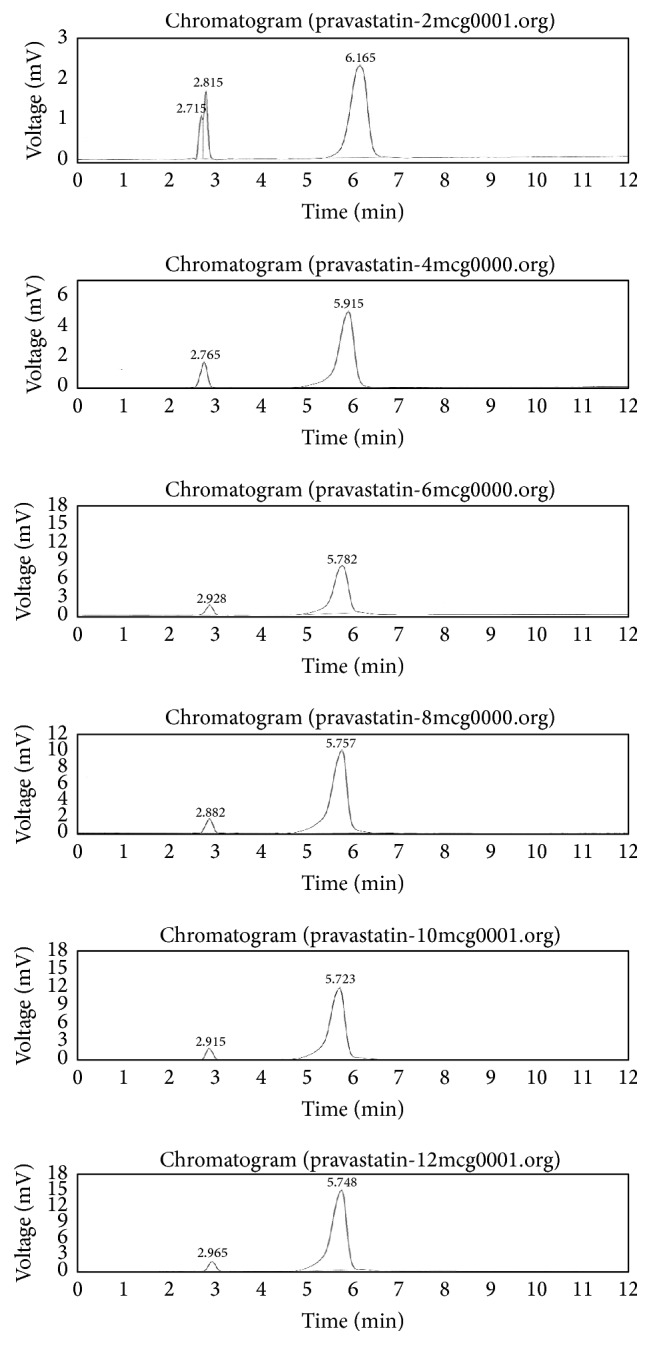
Standard calibration chromatogram by using HPLC method.

**Figure 8:**
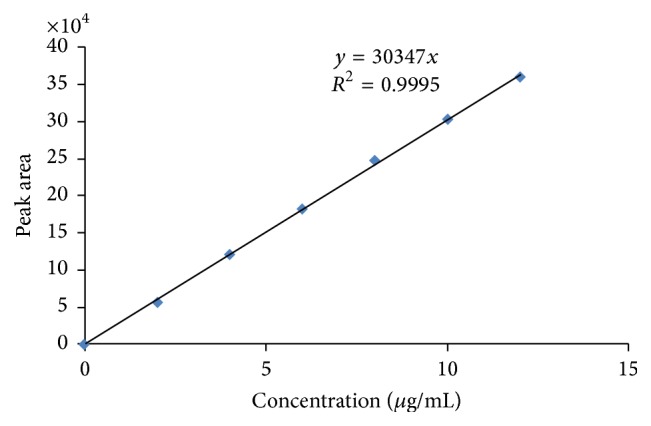
Calibration curve for Pravastatin Sodium by HPLC method.

**Figure 9:**
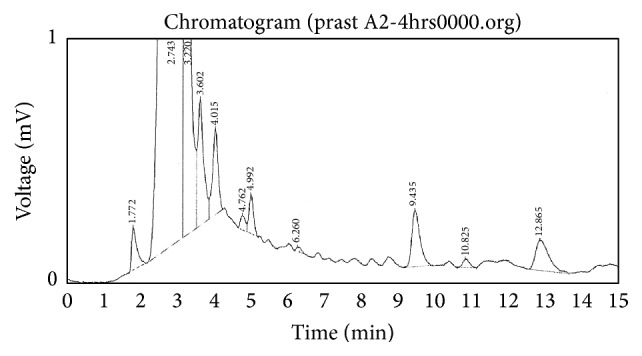
Determination of Pravastatin Sodium in rat Plasma by HPLC.

**Figure 10:**
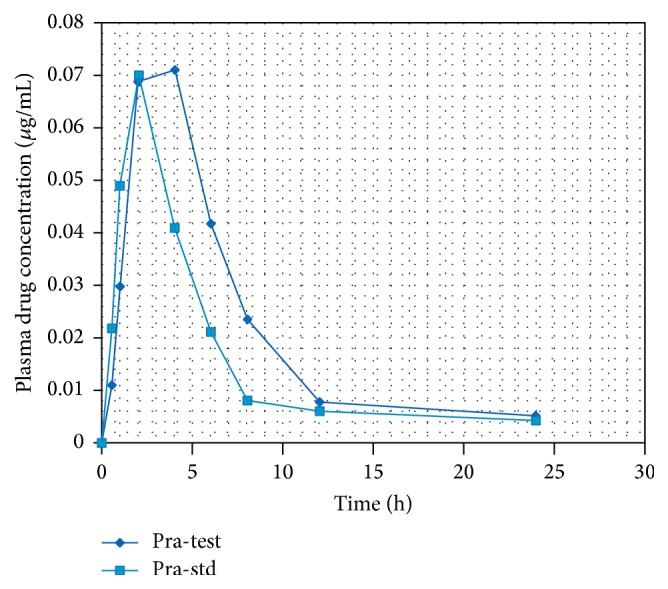
Time v/s plasma drug concentration AUC.

From HPLC method standard calibration data was prepared as shown in Table 4. Standard chromatogram of Pravastatin Sodium pure drug at different time intervals was as shown in Figure 7 and linearity was constructed as shown in Figure 8. Highest sharp peak at 6.205 in the chromatogram was observed. Pravastatin Sodium drug peak was observed at 6.280 in rat plasma as shown in Figure 9. Time versus average serum drug concentrations in microcapsules following oral administration in rats in both test and standard data at different time intervals were shown in Table 5. Time versus average serum drug concentration profiles (Figure 10), from which AUC pharmacokinetic parameters were calculated, were shown in Table 6. From area under the curve, we can calculate the extent of drug absorption after 24 h. *C*_max_ was observed to be 0.072 *μ*g/mL; *t*_max_ was observed to be 4 h. The *K*_el_ for Pravastatin Sodium microcapsules was observed to be 0.14/h and the resultant biological half-life was found to be 4.95 h. The mean residence time was observed at 6.3 h. Ka was found to be 0.57/h. When compared to standard about 2-fold increase in mean residence time was observed as shown in Table 6. After 6 months of intermediate stability studies there was no significant changes in % drug entrapment and* in vitro* drug release studies were observed as shown in Tables 7 and 8.

## 4. Summary and Conclusion

In case of formulation of Pravastatin Sodium microcapsules, Tulsion 412 ion exchange resins were complexed with the drug in different ratios for 4 h. Drug to resin ratio of 1 : 4 gave maximum amount of complexation achieved, 80.34%. Drug, resins, drug-resinates, and polymers interaction studies were carried out using FTIR, XRD, and DSC studies; there was no interaction observed between drug, resin, and polymers. Drug-resinates were further coated with Eudragit RSPO and Eudragit RLPO polymers for achieving control release in the small intestine. Microcapsules were prepared by using nonaqueous solvent evaporation method. Microencapsuled drug-resinates exhibited satisfactory values of angle of repose and bulk density. The drug content was found to be more than 96% and then subjected to evaluation studies like percentage yield, surface characteristics, and other physicochemical characteristics. Most satisfactory formulation f6 shows excellent physicochemical characteristics. Better drug retaining characteristics were observed in dissolution process.* In vitro* dissolution studies showed a drug release up to 75% in 8 h, which was found to be better drug retaining characteristics.* In vivo* bioavailability study was conducted by using rats. From pharmacokinetic evaluation, the mean residence time was found to be 6.3 h and indicates that more residence time was observed, thus indicating that Pravastatin Sodium microcapsules were released and absorbed slowly over a prolonged period of time. Further the formulations were subjected to stability testing for three months. Results revealed no significant changes in the formulations. The results from these studies demonstrated that microencapsules are a viable approach for developing a controlled release solid dosage form of Pravastatin Sodium. Drug-resinates coated with Eudragit RSPO (f6) have proved to be efficient carrier for diffusion controlled release microcapsules of Pravastatin Sodium. Optimized formulations provide better drug retaining characteristics; we can decrease the frequency of administration; it also avoids first pass metabolism and dose dumping; we can reduce the dose size and achieve better patient compliance.

## Figures and Tables

**Table 1:** Formulation chart.

Formulation code	Drug : resin ratio	Quantity of drug resinates	Eudragit RSPO (% w/w)	Formulation code	Drug : resin ratio	Quantity of drug- resinates	Eudragit RLPO (% w/w)
f1	1 : 4	1 g	—	—	—	—	—
f2	1 : 4	1 g	10%	f5	1 : 4	1 g	10%
f3	1 : 4	1 g	20%	f6	1 : 4	1 g	20%
f4	1 : 4	1 g	30%	f7	1 : 4	1 g	30%

**Table 2:** Physicochemical properties of developed formulations.

B-code	Drug-resinates to polymer ratio (% w/w)	% yield^*∗*^	Angle of repose (°)^*∗*^	Compressibility index^*∗*^ (%)	Bulk density^*∗*^ (g/cc)	% DEE^*∗*^	% coating of polymer^*∗*^	Particle size (*μ*m)
f1	—	97.0 ± 2.25	22.0	7.15	0.56	98.9 ± 0.5	97.4 ± 0.6	45–198
f2	10	95.1 ± 3.25	22.2	7.50	0.48	97.1 ± 0.8	97.6 ± 2.6	60–199
f3	20	96.3 ± 6.30	22.5	6.01	0.55	97.6 ± 0.8	97.1 ± 7.8	63–242
f4	30	97.9 ± 7.5	22.3	5.61	0.40	97.9 ± 0.8	98.7 ± 8.9	87–323
f5	10	98.1 ± 5.75	22.1	5.00	0.40	97.9 ± 0.5	97.9 ± 7.6	59–205
f6	20	98.8 ± 7.1	22.5	5.55	0.41	98.7 ± 0.3	98.6 ± 8.8	62–214
f7	30	97.4 ± 4.84	22.4	6.88	0.52	98.7 ± 0.2	98.9 ± 9.7	80–344

^*∗*^All the values were average mean of 3 determinations.

**Table 3:** Drug release kinetic data from microcapsules.

Batch	Zero-order *r* ^2^	First-order *r* ^2^	Higuchi equation *r* ^2^	Korsmeyer Peppas equation	
				*r* ^2^	*n*
f1	0.760	0.950	**0.976**	0.469	0.73
f2	0.716	0.974	**0.973**	0.462	0.71
f3	0.760	0.922	**0.901**	0.761	0.76
f4	0.710	0.917	**0.974**	0.481	0.76
f5	0.882	0.750	**0.989**	0.302	0.73
f6	0.911	0.736	**0.981**	0.897	0.59
f7	0.940	0.797	**0.970**	0.943	0.56

**Table 4:** Calibration data for pravastatin sodium by HPLC.

Serial number	Linearity-pravastatin sodium concentration (*μ*g/mL)	Peak area
1	2	57163.261
2	4	121376.268
3	6	182935.218
4	8	247918.698
5	10	303747.489
*6*	*12*	*360683.698*

**Table 5:** Average serum drug concentration in microcapsules following oral administration in rats.

Time (h)	Avg serum drug conc test (*µ*g/mL)	Avg serum drug conc Std (*µ*g/mL)
0	0	0
0.5	0.011 ± 0.0004	0.022 ± 0.003
1	0.030 ± 0.0002	0.049 ± 0.006
2	0.069 ± 0.002	0.07 ± 0.004
4	0.071 ± 0.001	0.041 ± 0.006
6	0.0417 ± 0.001	0.0213 ± 0.002
8	0.0236 ± 0.001	0.0081 ± 0.001
12	0.0079 ± 0.003	0.0061 ± 0.006
24	0.0052 ± 0.001	0.0043 ± 0.003

**Table 6:** Pharmacokinetic parameters.

Product	*C*_max_	*t*_max_	AUC	% relative BA	Elimination rate constant	Biological half-life	Vd	Ka	MRT	Cl
Test	0.072 *µ*g/mL	4 h	0.812 *µ*g/mL	90.38	0.14/h	4.95 h	16 L	0.57/h	6.3 h	0.293 h/mL
Std	0.07 *µ*g/mL	2 h	0.734 *µ*g/mL	—	0.15/h	2.9 h	12 L	0.50/h	3.5 h	0.251 h/mL

**Table 7:** Stability data of most satisfactory f6 formulation.

Parameter	Initial	After 3 months	After 6 months
Drug entrapment efficiency (%)	98.9 ± 0.1	98.4 ± 0.2	98.0 ± 0.1

**Table 8:** Dissolution data of formulation f6 after stability studies.

Time (h)	Initial	After 3 months	After 6 months
		30 ± 2°C/65 ± 5% RH	30 ± 2°C/65 ± 5% RH
	% CDR	% CDR	% CDR
1	24.6 ± 0.1	23.2 ± 0.3	22.0 ± 0.6
2	39.1 ± 0.9	38.7 ± 0.5	38.1 ± 0.1
3	45.0 ± 1.0	43.0 ± 0.1	42.8 ± 0.5
4	50.0 ± 0.1	49.8 ± 0.5	49.1 ± 0.7
5	51.8 ± 0.5	50.1 ± 0.2	49.0 ± 0.8
6	54.9 ± 0.4	54.0 ± 0.3	53.9 ± 0.7
7	69.3 ± 0.3	68.9 ± 0.7	68.0 ± 0.4
8	75.0 ± 0.2	74.9 ± 0.8	74.8 ± 0.3
